# The evolving role of radiotherapy in non-small cell lung cancer

**DOI:** 10.1259/bjr.20190524

**Published:** 2019-11-21

**Authors:** Sean Brown, Kathryn Banfill, Marianne C. Aznar, Philip Whitehurst, Corinne Faivre Finn

**Affiliations:** 1 The Christie NHS Foundation Trust, Manchester, UK, Manchester, UK; 2 Division of Cancer Sciences, Manchester Cancer Research Centre, Manchester Academic Health Sciences Centre, The University of Manchester, Manchester, UK, Manchester, UK; 3 Clinical Trial Service Unit, Nuffield Department of Population Health, University of Oxford, UK; 4 Christie Medical Physics and Engineering (CMPE), The Christie NHS Foundation Trust, Manchester Academic Health Sciences Centre, Manchester, UK

## Abstract

Lung cancer is the most commonly diagnosed cancer and biggest cause of cancer mortality worldwide with non-small cell lung cancer (NSCLC) accounting for most cases. Radiotherapy (RT) plays a key role in its management and is used at least once in over half of patients in both curative and palliative treatments. This narrative review will demonstrate how the evolution of RT for NSCLC has been underpinned by improvements in RT technology. These improvements have facilitated geometric individualization, increasingly accurate treatment and now offer the ability to deliver truly individualized RT. In this review, we summarize and discuss recent developments in the field of advanced RT in early stage, locally advanced and metastatic NSCLC. We highlight limitations in current approaches and discuss future potential treatment strategies for patients with NSCLC.

## Introduction

 Lung cancer is the most commonly diagnosed malignancy and the biggest cause of cancer mortality worldwide, accounting for 1.6 million deaths per year.^1^ Non-small cell lung cancer (NSCLC) makes up the majority of lung cancer cases. Treatment for NSCLC depends mostly on the stage of disease and patient fitness. Radiotherapy (RT) is used in all stages of lung cancer treatment and is required at least once in over half of patients for either cure or palliation.^2^

Historically, RT for lung cancer was planned in a simulator using parallel opposed fields and anatomical landmarks to define the target.^3^ The introduction of three-dimensional (3D) conformal RT using CT planning in the 1990s allowed improved tumour coverage and reduction in dose to organs at risk (OARs). Even more conformal treatment has become possible with the advent of intensity modulated radiotherapy (IMRT) in which the RT beam fluence, weight and shape are varied for multiple beams during treatment.^4^

Imaging capabilities have progressed alongside RT. Four-dimensional CT (4DCT), in which the respiratory motion of the tumour is visualized, has facilitated smaller margins individualized to a patient’s breathing cycle. This motion adaptation reduces the risk of a geographical miss in lung cancer RT.^5^ Cone beam CT (CBCT) has replaced two-dimensional megavoltage portal imaging to provide a more accurate set-up.^6^ These advances in imaging and RT technology allow individualized RT plans. Although the treatment dose, fractionation and dose limits for OARs do not change between patients, the dose distribution can be tailored to each individual’s anatomy.^7^ Whilst approaches to systemic therapy have become increasingly personalized according to tumour histology and molecular status,^8^ curative RT is still prescribed mainly according to the TNM stage, performance status and comorbidities, taking no account of the tumour biology. This narrative review will demonstrate how the evolution of RT for NSCLC has been underpinned by improvements in RT technology. This has facilitated geometric individualization, increasingly accurate treatment and now offers the ability to deliver truly individualized RT. In this review, we summarize and discuss recent developments in the field of advanced RT in early stage, locally advanced (LA) and metastatic NSCLC.

## Geometric individualiZation

**Figure 1.**
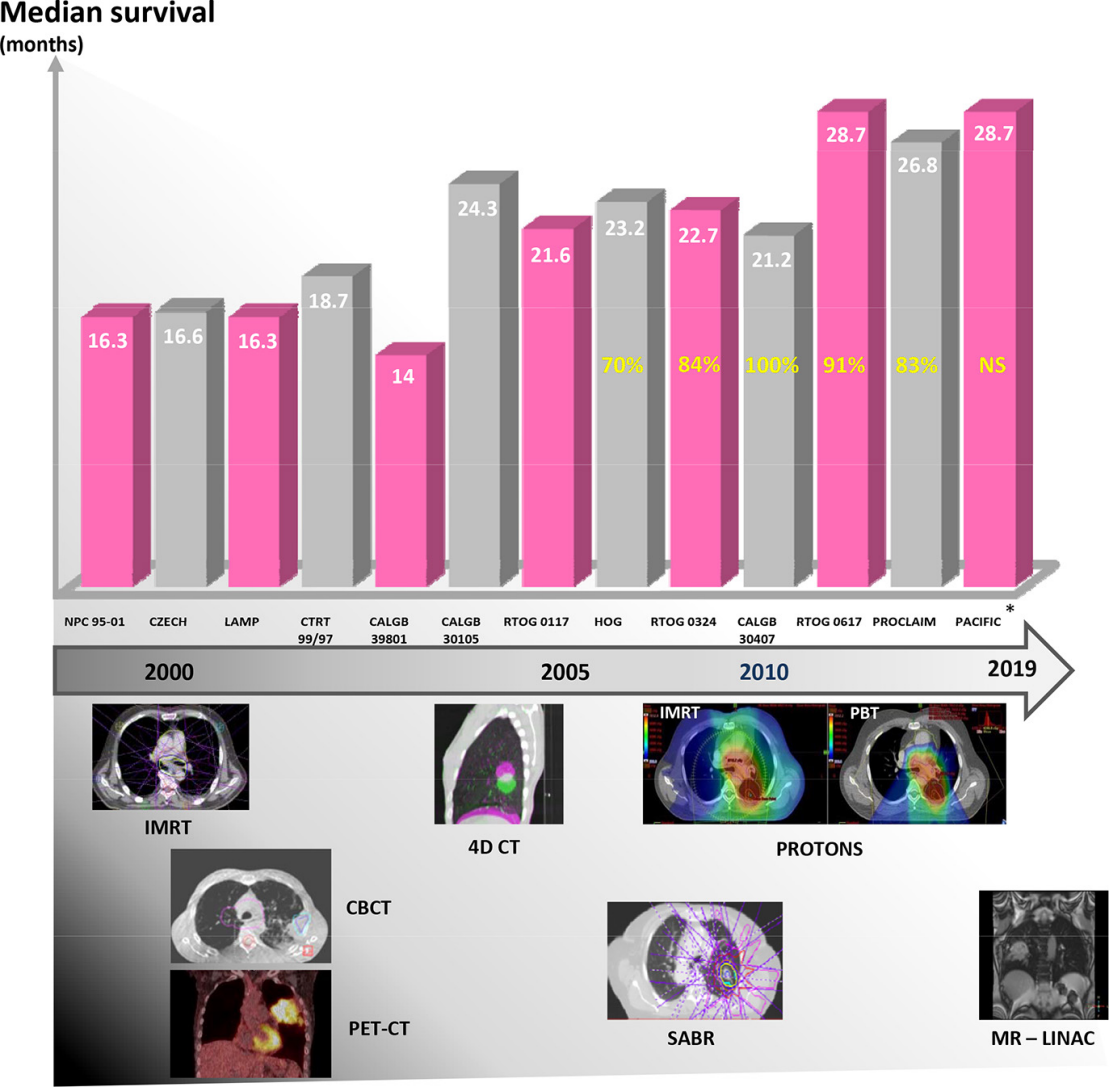
The improving (median) survival of patients with Stage 3 NSCLC (based on trial data). This is presented with a timeline showing the introduction of technologies that have been attributed to improving the accuracy of RT (note this does not necessarily correlate with implementation into the clinical workflow). Yellow numbers: percentage of patients within each trial staged with PET-CT, this information was NS by the PACIFIC investigators. *PACIFIC survival data: This is from the control arm only. The median figure has not yet been reached in the durvalumab arm. Note randomization occurred post chemoradiation. Therefore, survival is measured from this point onwards. 4DCT,four-dimensional CT; CBCT, cone beam CT; IMRT, intensity modulated radiotherapy;MR-linac, magnetic resonance linear accelerator; NS, not supplied; NSCLC, non-smallcell lung cancer; PET-CT, positron emissiontomography-CT; RT, radiotherapy; SABR, stereotactic ablative radiotherapy.

There are a number of challenges involved in the delivery of safe and accurate RT to the thorax.^9^ The lung has a low electron density. This can result in altered lateral dose, thus reducing the steepness of the dose fall-off and the conformality of the dose distribution. Physiological motion, tumour heterogeneity and the proximity of radiation sensitive healthy tissues, such as the lung, oesophagus and heart, increase the complexity of RT delivery. Historically, the lack of image guidance may have increased the likelihood of a geographical tumour miss, while simple beam arrangements and the uniform dose of large fields might have limited the potential of delivering a curative dose.^3^ Significant developments in advanced RT technologies have helped address these challenges.^10–13^ Figure 1 charts the development of RT technologies over time and the corresponding improvement in median survival in trials of patients with Stage 3 NSCLC.

The first of these developments to improve RT conformality was the introduction of the multileaf collimator (MLC), which shapes the RT field to the target. In addition to beam shaping, IMRT uses multiple MLC arrangements in order to provide fluence modulation. This enables sculpting of high doses around the target and away from OARs.^14^ Volumetric modulated arc therapy (VMAT), also called rotational IMRT, allows further modulation. This is achieved by varying the dose rate and enabling dynamic gantry rotation during beam delivery. In combination with a flattening-filter-free technique, VMAT can reduce treatment delivery time compared to fixed-beam IMRT.^15^ A shorter delivery time is particularly useful when delivering a high dose per fraction as in stereotactic ablative radiotherapy (SABR).^16^

As a result of the additional level of modulation compared to 3D conformal RT, IMRT can enhance the therapeutic ratio by optimizing dose to the tumour whilst sparing normal tissues. Consequently, IMRT will reduce toxicity and permit the radical treatment of patients with large tumours, who in the past would have been treated palliatively.^4^ Although there has not been a randomized trial comparing IMRT and 3D conformal RT for the treatment of lung cancer, some evidence for the use of IMRT is provided from a planned secondary analysis of data from the multi-institutional Phase III RTOG 0617 trial. This trial compared dose escalated chemoradiotherapy (CRT) with standard CRT. 47% of patients in this trial were treated with IMRT. Significantly more patients in the IMRT group had Stage 3B NSCLC and these patients had larger planning target volumes (PTVs) than those treated with 3D conformal RT.^12^ Despite this imbalance, which did not favour the IMRT group, there was no difference in survival between patients treated with 3D conformal RT, or IMRT. Furthermore, despite the larger PTVs, patients treated with IMRT had significantly less G3-5 pneumonitis and lower heart doses. This provides some evidence to support the use of IMRT and demonstrates how geometric individualization with IMRT can be used to treat patients with larger lung tumours.

4DCT constitutes another major technological development; it has helped to individualize RT planning by incorporating patient-specific information on respiratory motion.^17^ The patient’s respiratory cycle is recorded using a respiratory monitoring system, multiple axial CT images are then acquired throughout respiration and sorted into 8–10 phases ("bins").^5^ Each bin is then reconstructed as a 3DCT image representing the thoracic anatomy during a specific phase of the respiratory cycle. This process allows for accurate characterization of the displacement of the target volume (*e.g.* tumour and lymph nodes) and OARs due to breathing. 4DCT has improved treatment individualization by facilitating smaller margins and reducing the risk of a geographical tumour miss.^5^ The European Organization for Research and Treatment of Cancer and the European Society for Radiotherapy and Oncology Advisory Committee on Radiation Oncology Practice planning guidelines strongly recommend the use of a contrast-enhanced 4DCT for planning RT for lung cancer. These guidelines are summarized in Supplementary Material.^7,18^

On-treatment imaging [or image-guided radiotherapy (IGRT)] is important for correcting variations in patient set-up or anatomy between RT fractions, which can lead to a geographical tumour miss. The first forms of on-treatment imaging, *i.e*. kilo or megavoltage radiographs (portal imaging) could be used to match to anatomical surrogates (*e.g.* bone) but were often not sufficient to visualize the target. These approaches have largely been replaced by cone beam CT (CBCT) which provides better soft-tissue visualization, hence a more accurate set-up.^6^ If set-up errors are observed on the pre-treatment CBCT, the patient position is adjusted by shifting the couch before delivery. The importance of correcting set-up errors is underlined by recent evidence that uncorrected shifts, moving the high dose region towards the heart, were associated with worse survival.^19^ However, shifting the patient position is unable to account for changes in tumour shape and volume. As a result, the European Organization for Research and Treatment of Cancer guidelines recommend daily CBCT with soft tissue set-up to assess the presence of intrathoracic anatomical changes (ITACs) which may negatively impact upon the dose distribution.^7^ If these occur a new treatment plan, adapted to account for the impact of the ITAC should be created. This is known as reactive adaptation.^7^ Kwint et al have demonstrated that ITACs can occur in around 70% of patients undergoing curative radiotherapy.^20^ This was assessed predominantly in patients with Stage 3 disease (76%) and patients treated with SABR were excluded. They developed a decision support system to guide therapy radiographers in assessing the potential impact of ITACs upon the treatment and when to request clinician or physics support. Recent work has demonstrated that ITACs can occur in up to 22% of patients being treated with SABR.^21^ Most are minor but can be associated with unplanned clinician or physics review and significantly impact upon set-up time. A decision support may help identify SABR patients who may need replanning in order to avoid a geographical miss of the tumour and reduce the impact upon the workflow of clinically insignificant ITACs.

**Figure 2.**
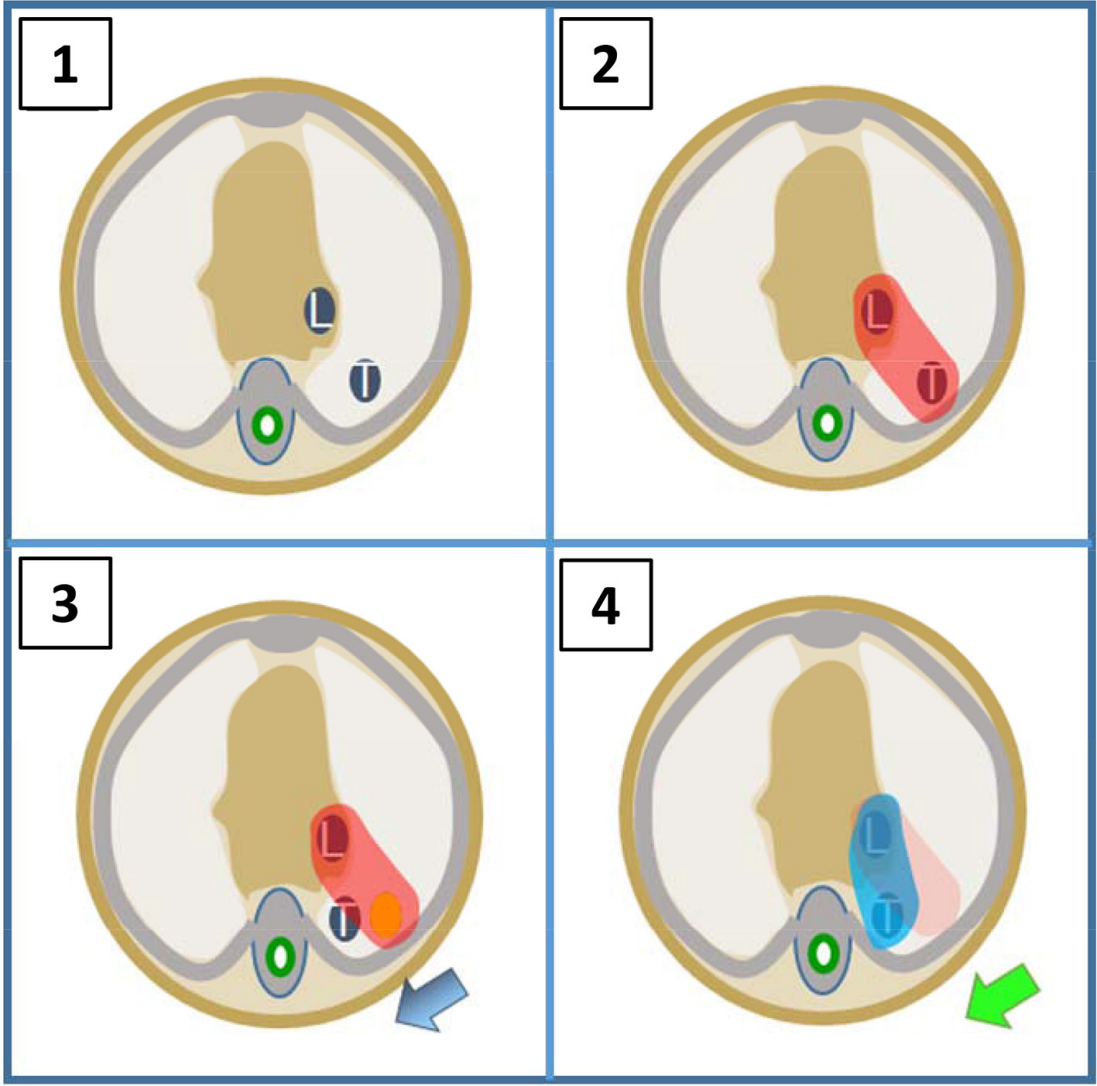
Treatment adaptation. (1) RTP CT demonstrates locally advanced lung cancer, tumour (T) and lymph node (L). (2) RTP CT with PTV covering T and L. (3) On-treatment image shows baseline shift causing tumour to move outside of PTV and high risk of geographical miss. (4) Treatment adapted to produce new PTV covering new position of L and T. PTV, planning target volume; RTP, radiotherapy treatment planning.

There is emerging interest in using on-treatment imaging to undertake proactive adaption in which potential ITACs are anticipated and the treatment plan adapted to account for changes in target geometry on a regular (even daily) basis (Figure 2). Such a strategy of frequent plan adaptation incorporated into fractionated RT could maximize tumour coverage, whilst minimizing the dose to OARs.^22^ There are however, concerns that reducing the target volumes in response to tumour regression may leave microscopic disease undertreated and increase the risk of local failure.^23^ This was assessed in the Phase 2 LARTIA trial where 217 patients with Stage 3 NSCLC were treated with CRT and underwent weekly chest CT. A new treatment plan was created if there was clinically significant tumour shrinkage and the treatment fields were reduced accordingly.^24^ In total, new plans were created in 23% of patients. While this trial showed encouragingly low rates of toxicitiy (2% acute and 4% late ≥Grade 3 lung toxicity) and acceptable numbers of marginal failures (6%), there was no consistent definition of what constituted clinically significant tumour shrinkage and therefore no objective criterion for the decision to create a new plan. Consequently, this approach should not be implemented in the routine setting until further validation is performed, ideally in the context of randomized studies. The MR-linac gives better target visualization compared to CBCT and provides the ideal platform to facilitate the investigation of daily online plan adaptation.^25^

## Early stage NSCLC

Conformal RT, 4DCT and IGRT have led to greater confidence in the accuracy of RT delivery and supported the development of SABR. This is a technique in which an ablative dose of RT is delivered with high precision in a small number of fractions. SABR is accepted worldwide as a standard of care in early stage NSCLC patients not suitable for surgery.^7,26,27^ In the UK, 54–60 Gy is usually delivered over 3–8 alternate day fractions.^28^

The CHISEL trial comparing SABR with radical fractionated RT in medically inoperable patients with Stage I NSCLC found that SABR is associated with better local tumour control at 2 years*—*89% compared to 65% [hazard ratio (HR) 0.32, 95% CI 0.13–0.77, *p* = 0.008].^29^ Moreover, SABR was found to improve the median overall survival (OS, 5 years *vs* 3 years*—*HR 0·53; 95% CI 0·30–0·94, *p* = 0·027) which is consistent with the results of retrospective series of SABR.^30^

SABR for peripherally located tumours is typically associated with low toxicity. This is, in part, due to the small volumes treated (maximum tumour size ≤5 cm) but also the highly conformal dose distribution and steep dose gradients that minimize irradiation of healthy tissues. However, in early series of SABR, patients with tumours within 2 cm of the proximal bronchial tree (PBT) treated with a biologically equivalent dose ≥210 Gy had a higher incidence of treatment related mortality.^31^ As a result, the recommendation is that these central lung tumours should be treated with more fractionated regimens such as 60 Gy in 8 fractions, provided the OAR tolerances are met.^32^ The rate of non-cancer death in patients who receive these risk-adapted schedules for SABR to tumours from 1 to 2 cm of the PBT has been found, in a large retrospective analysis, to be equivalent to that of patients who receive SABR to more peripheral tumours. Patients in this study with tumours within 1 cm of the PBT had a higher rate of non-cancer death, although the number of patients in this group was small.^33^ A recent Phase I study showed that the maximum tolerated dose in centrally located tumours treated with a five fraction regime is 12 Gy per fraction.^34^ The organs closest to the PTV, and therefore most at risk, were the main bronchus and the large vessels. There is a need for prospective clinical trials of SABR for central tumours in order to assess efficacy and define OAR constraints; Table 1 details ongoing SABR trials in centrally located early stage NSCLC. Unfortunately the LungTech trial, a multicentre Phase II trial in early stage NSCLC patients with central tumours, has closed early due to slow patient accrual. This was in part due to by two safety-related halts in recruitment.^35,36^

**Table 1.** Trials of SABR in central lung tumours

Trial	Study population	Study design	Primary outcome	Status
SUNSET NCT03306680^a^	Stage 1 NSCLC, ultra-central tumours –i.e. where PTV touches the central bronchial tree, great vessels or oesophagus	Phase I dose escalation study using a time-to-event continual re-assessment method Starting dose: 60 Gy in eight fractions Will escalate to 60 in five fractions (or de-escalate to 60 in 15 fractions if needed)	MTD *i.e*. dose associated with *a* < 30% rate of Grade 3–5 toxicity occurring within 2 years of treatment	Recruiting
LUNGTECH NCT01795521	Stage I-II NSCLC, centrally located in or abutting the 2 cm zone around the proximal bronchial tree and mediastinum),≤7 cm	Single arm Phase II study 60 Gy in eight fractions	Freedom from local progression	Closed early due to poor accrual
HILUS trial	Stage I-II NSCLC or progressive metastasis from another solid tumour, centrally located (≤1 cm from the proximal bronchial tree),≤5 cm	Single arm Phase II 56 Gy in eight fractions	Assessment of toxicity	Closed

NSCLC, non-small cell lung cancer; PTV, planning target volume; MTD, maximally tolerated dose

a ClinicalTrials.gov identifier

Clinical trials of SABR for ultracentral tumours, in which the PTV overlaps with central structures such as PBT, oesophagus or heart, are also required. In a Dutch retrospective study of 47 patients with ultracentral early stage NSCLC treated with SABR (60 Gy in 12 fractions), treatment-related deaths were reported in 21% of patients including fatal pulmonary haemorrhage in 15%.^37^ There is therefore an unmet need to define OAR constraints in such a clinical scenario.

While SABR has been shown as the best treatment for early stage lung cancer in people for whom surgery is not an option due to medical comorbidities, the role of SABR in those who are fit for surgery is more controversial. Retrospective analyses have shown that surgery and SABR have equivalent cancer specific survival.^38–40^ Patients who have surgery have improved OS, however, this is partly because these patients are younger and have fewer comorbidities. Clinical trials of SABR *vs* surgery for peripheral tumours are shown in Table 2. Some of these have closed early due to poor recruitment (highlighted pink) as patients and physicians often have a preference for one treatment modality. The UK SABR-TOOTH trial attempted to remove this lack of equipoise by having the diagnosing respiratory team discuss and consent patients with borderline operable early stage NSCLC to the trial.^41^ Following randomization, the patient was then reviewed by either the surgeon or the clinical oncologist. Despite this study design, the trial also failed to recruit patients within the predefined recruitment timelines. It is hoped that other ongoing randomized trials will provide clarity on the question of surgery or SABR for early stage lung cancer.

**Table 2.** Trials of SABR *vs* surgery for early stage NSCLC

Trial	Study population	Study design	Primary outcome	Status
ROSEL NCT00687986^a^	Stage 1A NSCLC,>2 cm from PBT, fit for surgery	Phase III **Arm A:** SABR (60 Gy in three or five fractions) **Arm B:** Primary surgical resection	Local and regional control	Closed early due to poor accrual
STARS NCT00840749	Stage 1A/B NSCLC, fit for surgery	Phase III **Arm A:** SABR (60 Gy in four fractions if central or three fractions if peripheral using Cyberknife) **Arm B:** Surgery	OS	Closed early due to poor accrual
ASOSOG-RTOG NCT01336894	Stage 1 NSCLC, fit for surgery	PhaseIII **Arm** **A:** SABR (3 fractions) **Arm B:** Sub-lobar resection+/- intra-operative brachytherapy	OS	Closed early due to poor accrual
SABR-TOOTH ISRCTN13029788^b^	Stage I NSCLC, peripheral tumours, patients at higher risk from surgery	Phase II feasibility study **Arm A:** SABR **Arm B:** Surgery	Recruitment rate	Closed early due to poor accrual
STABLE-MATES NCT02468024	Stage 1 NSCLC (≤4 cm), high risk operable patients	Phase III, patients are randomized before they consent to trial **Arm A:** SABR (54 Gy in three fractions) **Arm B**: Sub lobar resection	OS	Recruiting
POSTILV NCT01753414	Stage 1 NSCLC (≤3 cm), fit for surgery	Randomized Phase II **Arm A:** SABR (55 gy in five fractions) **Arm B:** Complete surgical resection	Local-regional control	Recruiting
VALOR NCT02984761	Stage 1 NSCLC (≤5 cm), includes central tumours (≥1 cm from trachea, oesophagus, brachial plexus, first bifurcation of the PBT, or spinal cord) fit for surgery	Phase III **Arm A:** SABR (54 Gy in three fractions, 56 Gy in four fractions or 57.5 Gy in five fractions. Central tumours will receive 50 Gy in five fractions) **Arm B:** Anatomic pulmonary resection	OS	Recruiting

NSCLC, non-small cell lung cancer; OS, overall survival; PBT, proximal bronchial tree; SABR, stereotactic ablative radiotherapy.

a ClinicalTrials.gov identifier

b ISRCTN.com identifier

## Locally advanced NSCLC

The current standard of care for treating fit patients with inoperable LA NSCLC is concurrent CRT.^42^ While improved imaging and RT technology have allowed the development of ablative doses for early stage NSCLC, the RT dose fractionation in LA-NSCLC has changed very little for over 30 years.^43^ Nevertheless, as shown in Figure 1, survival for patients with LA-NSCLC has improved over this time. One reason for the improved survival of these patients is better staging prior to treatment with positron emission tomography CT (PET-CT) and endobronchial ultrasound transbronchial needle aspiration.^18^ PET-CT has been demonstrated to upstage 24% of LA-NSCLC cases to Stage 4.^44^

Radiobiological evidence suggests that in NSCLC the delivery of higher radiation doses results in improved tumour control probability.^45^ Therefore, in the late 1990s and early 2000s, multiple Phase I/II RT dose escalation trials were performed in patients with surgically inoperable lung cancer in an attempt to improve patient survival.^46–51^ These led to a randomized Phase III trial (RTOG 0617) of CRT comparing 60 Gy and 74 Gy RT with or without cetuximab in patients with Stage 3A/B disease. This trial found that the median survival for patients in the dose escalated arm was worse than for the 60 Gy arm (20.3 months *vs* 28.7 months).^52^ Nevertheless, as shown in Figure 1, patients in the standard dose arm of RTOG 0617 had better survival than those in previous trials. This may partly be due to stage migration as 91% of patients in RTOG 0617 had a staging PET-CT. The reasons behind the poor survival of patients in the high dose arm have been extensively discussed and are summarized in box 1.^53,54^

Box 1. **Summary of reasons for the poor survival of patients in the 74 Gy high dose arm of RTOG 0617^52^**

**Figure:**
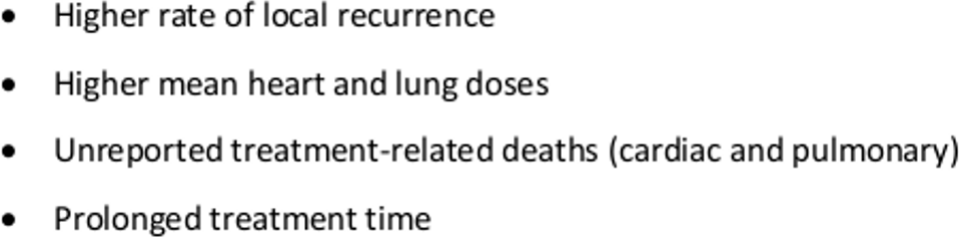

Although RTOG 0617 failed to demonstrate the benefit of dose escalation with conventional fractionation, a more individualized strategy deserves to be investigated. For example, functional imaging information can be used to increase the RT dose to particular areas. This is being used in the PET-boost trial, detailed in Table 3. Early data from the study report higher acute and late toxicities compared to standard CRT.^55^ Five cases (4.7%) of Grade 5 pulmonary haemorrhage have been observed in patients with central tumours encasing the vasculature; suggesting caution may be required in this scenario.

**Table 3.** the inTrials of individualiszed RT

Trial	Study population	Study design	Primary outcome	Status
**Adaption based on functional imaging**				
RTOG 1106 NCT01507428^a^	PET-CT avid (SUV ≥4) inoperable stage 3 NSCLC	Randomized Phase II **Arm A**: SOC CRT (60 Gy in 30 fractions) **Arm B:** Daily CRT over 30 fractions. Individualized adaptive RT; 2.4–3.5 Gy daily to MLD 20 Gy (≤86 Gy), based on PET-CT between fractions 18/19 Both arms will receive 3 cycles of consolidation chemotherapy (carboplatin/paclitaxel)	Local regional progression free rate Relative change in SUV peak from the baseline to the during-treatment PET-CT to local regional progression free rate	Closed
PET boost NCT01024829	Stage IB-III NSCLC, SUVmax on pre-treatment PET-CT ≥5	Randomized Phase II Stage IB-II patients receive RT alone, stage 3 patients receive sequential or concurrent CRT **Arm A:** 66 Gy in 24 daily fractions with integrated boost (≤72 Gy) to primary tumour **Arm B:** 66 Gy in 24 daily fractions with integrated boost (≤72 Gy) to the 50% SUVmax area of the primary (of pre-treatment PET-CT)	Local PFS	Closed
IFCT14-02/RTEP7	Inoperable locally advanced NSCLC	Randomized Phase II **Arm A:** Individualized RT ≤74 Gy in 33 fractions if a PET-CT at 42 Gy is positive Initial dose of 50 Gy will be given in 5 weeks (2 Gy daily), then ≤24 Gy in 1.6 weeks (bd fractions) **Arm B:** 66 Gy in 33 daily fractions with no adaptation Both arms will undergo 2 cycles of induction and subsequent concurrent platinum based chemotherapy	Local regional control rate	Recruiting
High Intensity Functional Image Guided VMAT Lung Evasion (HI-FIVE) NCT03569072	Stage 3 NSCLC	Single arm interventional pilot study (20pts) 60 Gy in 30 fractions with simultaneous integrated boost to primary tumour to 69 Gy RT adapted using ventilation/perfusion PET-CT to avoid regions of functional lung and boost tumour	To assess the technical feasibility of the delivery of personalized functional lung radiotherapy (*Treatment will be considered feasible if all of the following criteria are met: Reduction in mean functional lung dose of ≥2%, functional lung volume receiving 20 Gy of ≥4%, Mean heart dose is ≤30 Gy and relative heart volume receiving 50 Gy is <25%*)	Recruiting
FLARE RT NCT02773238	Inoperable stage IIB-IIIB NSCLC, PS 0–1	Single arm Phase II CRT with functional lung avoidance based on SPECT-CT. RT dose escalation if no response on PET-CT after 3 weeks	OS (in the functional lung avoidance and response-adaptive dose escalation RT cohort will be compared to 60 Gy cohort from RTOG 0617)	Recruiting
**Individualized RT**				
N12HYB NCT01933568	Inoperable stage II or III NSCLC Peripheral tumour <5 cm	Phase I Combined SABR to primary tumour and CRT to lymph nodes	Mean lung dose associated with a 15% probability of DLT;≥Grade three radiation pneumonitis and radiation induced dyspnoea	Closed
Hypofractionated IGRT NCT01459497	Stage II-III NSCLC, PS ≥ 2, PS0-1 and >10% wt loss or unfit for combined modality RT	Phase III **Arm A:** 60 Gy in 15 daily fractions with daily IGRT **Arm B:** 60–66 Gy in 30–33 daily fractions	OS	Recruiting
ADSCAN ISRCTN47674500^b^	Stage 3 NSCLC patients to be treated with sequential CRT	Randomized Phase II **Arm A** Standard RT arm 55 Gy in 20 daily fractions (2.75 Gy per fraction) **Arm B** CHART-ED, 54 Gy in 36 fractions (3 × 1.5 Gy fractions per day)+10.8 Gy in six fractions (days 15–17) **Arm C** IDEAL, 30 daily fractions of isotoxic RT, 63–71 Gy **Arm D** I-START**,** 20 daily fractions of isotoxic RT, 55–65 Gy **Arm E** Isotoxic IMRT, bi-daily fractions of isotoxic RT over 4 weeks, max dose 79.2 Gy	PFS	Recruiting

CRT, chemoradiotherapy; DLT, dose-limiting toxicity; MLD, mean lung dose; NSCLC, non-small cell lung cancer;PET-CT, positron emission tomography-CT; PFS, progression-free survival; RT, radiotherapy; SABR, stereotactic ablative radiotherapy; SOC, standard of care; SPECT, single-photon emission computed tomography; SUV, standard uptake volume; SUVmax, maximum standard uptake volume; VMAT, volumetric-modulated arc therapy.

a ClinicalTrials.gov identifier

b ISRCTN.com identifier

Another example of individualized dose-escalation is the delivery of RT based on the maximum dose that can be tolerated by the OARs (so called "isotoxic RT"). Studies have investigated this approach both in the sequential and the concurrent CRT setting.^56,57^ This led to a feasibility study in the UK (Isotoxic IMRT) showing a median tumour dose of 77.4 Gy (61.2–79.2 Gy; two fractions per day) can be delivered in LA-NSCLC treated with sequential CRT and IMRT.^58,59^ Isotoxic IMRT has been incorporated into the ongoing UK randomized Phase 2 ADSCAN trial comparing standard of care sequential CRT with three other dose escalated regimes in patients not suitable for concurrent CRT.^60^ The most efficacious RT regimen will then be compared to the standard of care in a Phase 3 trial. Other studies have looked at individualizing treatment by using ablative doses to boost residual or recurrent disease in patients with LA-NSCLC. However, this approach requires caution; data from four studies evaluating this technique show 5/80 (6.3%) patients experienced fatal toxicities such as haemorrhage.^61–64^ This was most notable when boosting centrally located disease and is similar to the outcomes observed when using primary SABR to treat central tumours.^65^ This and other ongoing trials examining ways to individualize dose escalated RT in NSCLC are shown in Table 3.

In many of these studies of isotoxic RT, the dose escalation is constrained by normal tissue tolerances of surrounding organs such as the oesophagus, lungs, airways and heart. These tissue tolerances are based on preclinical and clinical studies and have been synthesized in the Qualitative Analyses of Normal Tissue Effects in the Clinic.^66^ At present, the same dose volume constraints are used in all patients with little regard to patient factors, such as pre-existing comorbidities, smoking status, baseline lung or cardiac function, which could affect tissue tolerances.

Between 66 and 76% of patients with LA-NSCLC have at least one concomitant medical condition.^67,68^ Patients with comorbidities are often not included in clinical trials. Only 41% of patients on the Maastrict cancer registry would have met the inclusion criteria for RTOG 0617 due to comorbidities and no patient over the age of 75 would have been eligible.^69^ Trials which investigate RT in unfit or older patients (Table 3), are therefore important in attempting to answer questions about individualizing RT based on patient factors.

Despite the improvements in RT over the last 20 years, the largest improvement in survival of patients with inoperable LA-NSCLC has come, not through RT, but immunotherapy (IO). The addition of 1 year of durvalumab following CRT has been shown to improve 2 year OS (66.3% with durvalumab *vs* 55.6 with placebo) and progression-free survival (PFS) (median, 28.3 months *vs* 16.2 months).^70^ Such consolidation treatment is now considered standard of care in fit patients with LA-NSCLC who have responded to definitive concurrent CRT.^27^ In the UK, durvalumab is approved by the National Institute for Health and Care Excellence only in patients treated with concurrent CRT and with PDL1 >1%.^71^ Questions remain about the best way of integrating targeted therapies and RT in LA patients with driver mutations.

## Metastatic NSCLC

Traditionally, patients with Stage 4 disease received chemotherapy with a platinum doublet. Over the last decade, the treatment of this group of patients has become increasingly tailored to tumour histology, and outcomes have improved. Targeted agents are used in patients with tumours with molecular drivers such as EGFR, ALK and ROS-1 mutations. More recently, IO has transformed the treatment of metastatic NSCLC.^8^ Palliative RT for Stage 4 disease is used to improve symptoms such as pain, cough or haemoptysis.^72^ In recent years the concept of oligometastatic disease (OMD) has emerged and there is evidence that RT can improve survival in these patients.^73^ OMD is defined differently depending on the point in the disease process the distant disease is diagnosed (Table 4). The first evidence for the benefit of RT in OMD came from a randomized trial of whole brain radiotherapy with or without stereotactic brain RT in patients with 1–3 brain metastases. Over half of the patients in this trial had brain metastases from NSCLC. OS was significantly improved with the addition of stereotactic brain RT in patients with a single brain metastasis (6.5 months *vs* 4.9 months).^74^

**Table 4.** Trials in OMD

Type of OMD	Trial	Study population	Study design	Primary outcome
**SYNCHRONOUS** OMD is detected at diagnosis of the primary tumour 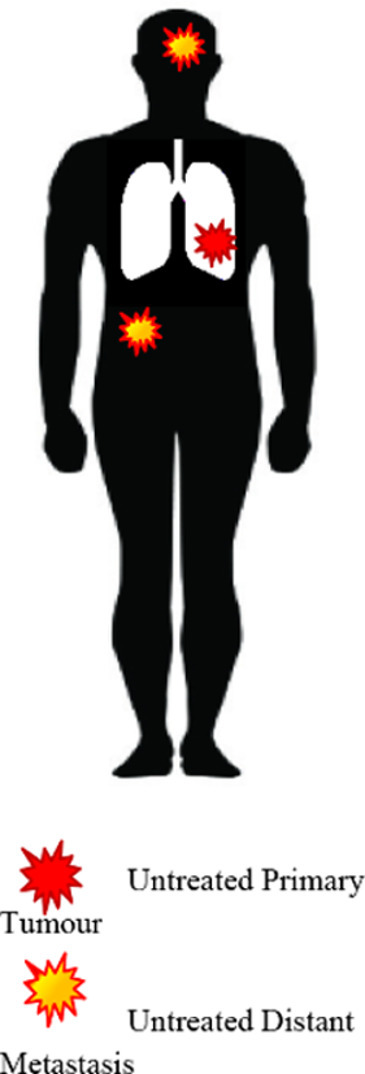	**SARON** NCT02417662^a^	NSCLC with ≤3 synchronous oligo-metastases^a^	Phase III **Arm A:** Standard treatment, chemotherapy alone **Arm B:** Chemotherapy followed by radical RT (SABR or conventional RT) to the primary and SABR to metastases	OS
	**NRG-LU002** NCT03137771	NSCLC with ≤3 metastases with no progression after four cycles chemotherapy	Randomized Phase II/III **Arm A:** Maintenance SACT only **ARM B:** Local consolidative therapy (LCT- SABR, IMRT, 3DCRT or possibly surgery) followed by maintenance SACT	Phase II: PFS Phase III: OS
	**LONESTAR** NCT 03391869	Metastatic NSCLC	Phase III **Arm A:** Nivolumab and ipilimumab alone **Arm B:** Nivolumab +ipilimumab and LCT (radiotherapy ± surgical resection, radiofrequency ablation or cryoablation)	OS in overall population OS in OMD (subgroup ≤3 lesions)
**METACHRONOUS** Oligo-recurrent OMD is detected after treatment of the primary tumour 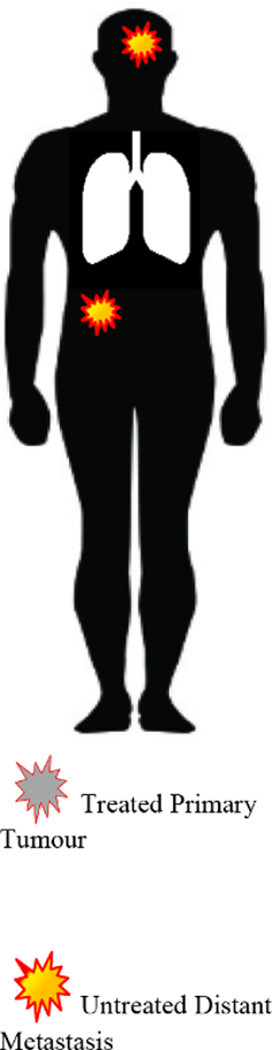	**CORE** ISRCTN45961438**˜**	Patients with NSCLC, breast or prostate cancer (previously completed radical treatment) with ≤3 extra cranial metastases suitable for SABR	Randomized Phase II/III **Arm A:** SABR to metastases and standard of care treatment (*e.g.,* SACT, palliative RT or observation) **Arm B:** Standard of care	PFS
	**SABR-COMET 10** NCT03721341	Patients with 4–10 new metastases from any primary tumour (who completed radical treatment for any >3 months previously) and have stable disease at primary site	Phase III **Arm A:** SABR plus standard of care treatment (SACT or observation) **Arm B:** Standard of care	OS
**OLIGO-PROGRESSIVE** OMD that occurs after response to systemic therapy. Progression of a limited number of areas of disease occurs while all other tumours remain controlled 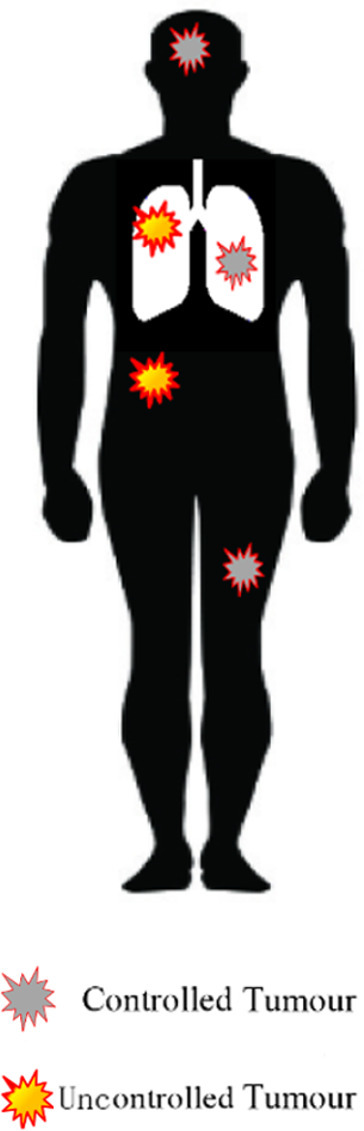	**HALT** ISRCTN53398136	Metastatic NSCLC with actionable mutation receiving TKI therapy, ≤3 extracranial sites of progressive disease	Randomized Phase II **Arm A:** SABR to progressive lesions and continuation of TKI **Arm B:** Continuation on TKI	PFS
	**STOP-NSCLC** NCT02756793	Metastatic NSCLC with actionable mutation receiving TKI therapy, ≤5 extracranial sites of progressive disease	Randomized Phase II **Arm A:** SABR to progressive lesions + continuation of TKI **Arm B:** Continuation on TKI	PFS
**OLIGO-PERSISTENT** This is a scenario where there are a limited number of controlled persistent lesions following a response to systemic therapy 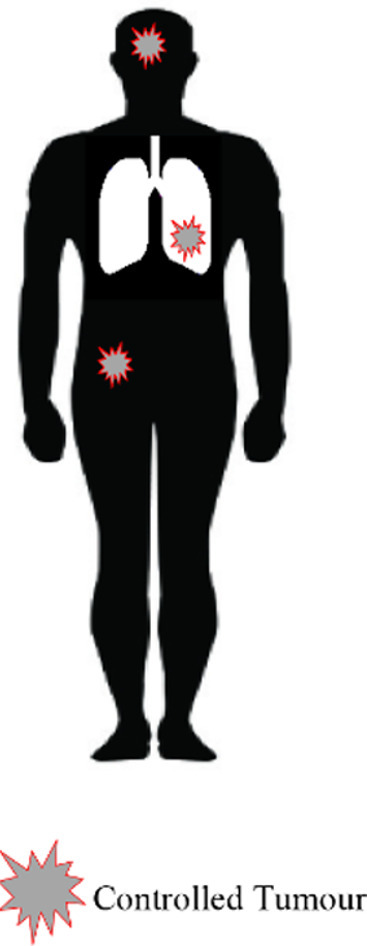	**NORTHSTAR** NCT03410043	EGFR mutant metastatic NSCLC, treated with 6–12 weeks of osimertinib, and oligo or poly persistent disease	Randomized Phase II **Arm A:** LCT (RT or surgery ± RT) and continue osimertinib **Arm B:** Osimertinib only	PFS

3DCRT, three-dimensional conformal radiation therapy; EGFR, epidermal growth factor receptor; IMRT, intensity modulated radiotherapy; NSCLC, non-small cell lung cancer;OMD, oligometastatic disease; OS, overall survival; PFS, progression-free survival; RT, radiotherapy; SABR, stereotactic ablative radiotherapy; SACT, systemic anticancer therapy; TKI, tyrosine kinase inhibitor.

a ClinicalTrials.gov identifier, ˜ISRCTN.com identifier

More recently, evidence is emerging for the use of SABR to treat extracranial metastatic disease in NSCLC from a number of randomized Phase II studies. Iyengar et al compared maintenance chemotherapy alone to SABR and maintenance chemotherapy in 29 patients with OMD from NSCLC. This was stopped early due to a significant improvement in PFS in the SABR arm (9.7 months *vs* 3.5 months).^75^ Another similar study of local therapy (including SABR, intermediate hypofractionated-RT, CRT or surgery) versus standard of care following systemic anticancer therapy was also stopped early for the same reason.^76^ Subsequently, an updated analysis of this trial reported an OS benefit with local therapy of 24 months.^77^ A larger randomized trial of 99 patients with any primary tumour (18% of whom had NSCLC) found improved OS with SABR to between 1 and 5 metastases (28 *vs* 41 months).^73^ Although the results of these trials are encouraging, the numbers of patients with OMD from NSCLC included are small and there are important ongoing questions regarding patient selection. Consequently, randomized Phase III evidence is required. There are many ongoing studies examining radiotherapy including both metastatic NSCLC and other cancer sites, shown in Table 4.

## Future developments

Over the past two decades radiotherapy has evolved into a highly precise treatment. This has led to an improvement in patient outcomes such as that seen with SABR for early stage disease, however in LA disease there is still an unmet need to improve results. Undoubtedly, future progress in lung cancer RT will come from the individualization of our advanced treatment strategies and the better integration with systemic therapies, including IO.

The MR-guided linear accelerator (MRL) and proton beam therapy both represent exciting developments in RT technology which may further advance targeting and individualization of treatment.

The MRL combines a linear accelerator with on-board diagnostic quality MRI. The superior soft tissue discrimination of MR gives better target visualization compared to CBCT. The improved target visualization may facilitate the investigation of daily online plan adaptation and isotoxic dose intensification without the concerns of excessive ionizing radiation that exist with CBCT.^22,25,59^ The MRL will also enable real-time gating and permit assessment of delivered dose compared to planned dose.^78^ Ultimately, the anatomical and dosimetric information obtained in real time may facilitate intrafractional plan adaptation, to ensure the intended daily dose is delivered. This should permit smaller PTV margins and further reduce the dose to healthy tissues. Therefore, MR-guided RT potentially represents truly individualized RT with a move away from an image-guided to a "dose-guided" technique.^22^ The MRL also offers the potential to incorporate biological information via functional imaging. This may enable the exciting possibility of individualized adaptation and intensification based on tumour metabolic activity and hypoxia.^79^

Proton beam therapy (PrBT) is another technology that has the potential to improve the outcome of NSCLC patients. The physical characteristics of protons mean their energy deposition increases with increasing depth in tissue. As a result, the maximum energy deposition occurs toward the end of the proton range (the Bragg peak). Therefore, the dose to the normal tissues beyond this range and the integral dose are reduced. A trial of IMRT *vs* passive scattering PrBT in patients with LA-NSCLC found that PrBT reduced low dose bath to the lungs although more lung tissue was exposed to doses ≥ 20 Gy. Moreover, PrBT spared more heart volume at all dose levels compared to IMRT.^80^ In spite of this dosimetric advantage, this randomized Phase III trial did not show a clinical benefit for patients treated with protons. There are many unsolved technological challenges in delivering PrBT to mobile targets such as lung tumours, as the sharp dose gradient of proton plans makes them particularly sensitive to geometric uncertainties. There are a number of studies ongoing in early and LA lung cancer patients comparing IMRT with PrBT (ClinicalTrials.gov:NCT 01993810, NCT 02731001 and NCT 03818776).

The addition of consolidation IO after concurrent CRT has recently become standard of care in LA NSCLC.^70^ There are currently many ongoing trials investigating a similar strategy in patients with early stage NSCLC and in LA patients not suitable for concurrent CRT. Clinical trials are also investigating the addition of IO to thoracic RT concurrently in combination with consolidation treatment. Many questions remain about the timing of the administration of IO and thoracic RT and the impact of the volume of irradiation on the immune system. Further research is also required to identify the patients most likely to benefit from consolidation IO. One other promising area of investigation is the concept of "minimal residual disease" with circulating tumour deoxyribonucleic acid (ctDNA).^81^ ctDNA represents an attractive biomarker and may have benefits in screening, assessing response to treatment and surveillance. Blood collection for circulating tumour cells and ctDNA is included in some of the trials of RT in OMD (Table 4).

## Conclusion

In this review, we have described the advances in imaging, tumour localization and RT technology that have facilitated the development of SABR for early stage NSCLC and allowed the treatment of large volume LA NSCLC. We have also highlighted ongoing research in areas such as dose escalation in LA disease and the use of SABR in the treatment of oligometastatic disease.

Advances in RT technologies have improved the precision of RT but in order to make further progress, there is an unmet need to tailor treatment to individual patients and tumour characteristics.
